# Growth Performance and Enzymatic Response of the Grasshopper, *Calliptamus abbreviatus* (Orthoptera: Acrididae), to Six Plant-Derived Compounds

**DOI:** 10.1093/jisesa/ieaa049

**Published:** 2020-06-05

**Authors:** Yueyue Wang, Xunbing Huang, Babar Hussain Chang, Zehua Zhang

**Affiliations:** 1 Shandong Provincial Key Laboratory of Water and Soil Conservation and Environmental Protection, College of Resources and Environment, Linyi University, Linyi, P.R. China; 2 State Key Laboratory of Biology of Plant Diseases and Insect Pests, Institute of Plant Protection, Chinese Academy of Agricultural Science, Beijing, P.R. China; 3 Department of Entomology, Sindh Agriculture University, Tando Jam, Pakistan

**Keywords:** plant-derived compound, grasshopper, enzyme activity, growth performance, biological control

## Abstract

Plant-derived compounds are sources of biopesticides for the control of insect pests. We compared the growth performance and enzymatic response of the grasshopper *Calliptamus abbreviatus* Ikonn to six plant-derived compounds (rutin, quercetin, nicotine, matrine, azadirachtin, and rotenone) in laboratory and field trials. When exposed to the six compounds, *C. abbreviatus* had significantly reduced growth and survival. All the compounds significantly induced an elevated level of reactive oxygen species, indicating oxidative damage. The activity of detoxifying enzymes, including cytochrome P450s, carboxylesterase, glutathione-*S*-transferase, and UDP-glucuronosyltransferase, and the antioxidant enzymes, including superoxide dismutase, catalase, and peroxidase, all significantly increased after exposure to the six compounds. These data suggest that the six plant-derived compounds had negative effects on *C. abbreviatus*. Of the six compounds, matrine, azadirachtin, and rotenone were more toxic to *C. abbreviatus*, followed by nicotine, quercetin, and rutin. These results show the potential of these compounds as botanical pesticides, which can be applied for the biological control of the grasshopper *C. abbreviatus*.

Chemical compounds extracted from plants have pest control potentials (Liu et al. 2006). The use of botanical compounds for pest control has increased in recent decades (Talukder 2006, Tangtrakulwanich and Reddy 2014). When used in integrated pest management programs, botanical pesticides generally have the advantage of short residual lives and do not accumulate in the environment (Khater 2012, Senthil-Nathan 2013). Some plant-derived alkaloids, phenolics, and terpenoids have long histories of use in insect control (Luo and Zhang 2003, Leatemia and Isman 2004, Liu et al. 2006, Khan et al. 2017), and consequently, these botanical insecticides are important in some plant protection programs.

Many plant extracts have been evaluated for their activity against agricultural insect pests (Haseeb et al. 2004, Xu et al. 2004, Akhtar et al. 2008, Taggar and Gill 2016). For example, nicotine, matrine, retenone, rutin, or quercetin extracts can cause sublethal, lethal, or other deleterious effects on many coleopteran and lepidopteran pest species. These species include *Pieris rapae*, *Plutella xylostella*, *Sitophilus zeamais*, *Lymantria dispar*, *Spodoptera litura*, *Pectinophora gossypiella*, *Heliothis virescens*, *Spodoptera eridania*, and *Helicoverpa zea* (Zeng et al. 2002, Poreddy et al. 2015, Zanardi et al. 2015, Chen et al. 2017). Pyrethrum is also a repellent and can modify insect behavior (Kessler and Baldwin 2002, Tan and Luo 2011, Senthil-Nathan 2013), and azadirachtin is a tetranortriterpenoid with ecdysis disrupting, antifeedant, and reproduction-inhibiting properties against more than 200 insect species (Liang et al. 2003, Liu et al. 2006, Tangtrakulwanich and Reddy 2014). In addition, azadirachtin can also produce intestinal lesions, induce reactive oxygen species (ROS) generation, affect the growth and development, and lead to death in some insects (Mordue 2004, Ley 2005, Huang et al. 2013, Senthil-Nathan 2013). Moreover, resistance to these botanical compounds has rarely developed in the field compared with conventional synthetic pesticides (Manyangarirwa et al. 2006, Tangtrakulwanich and Reddy 2014). This suggests that they would be effective tools in integrated pest management (IPM) for decreasing the use of conventional synthetic pesticides (Tangtrakulwanich and Reddy 2014). These effective toxic compounds, derived from plants, make a great contribution to the ‘natural’ control of insect pests.

However, some insects have developed metabolic resistance, target-site resistance, penetration resistance, and behavioral resistance to protect themselves from toxins (Després et al. 2007, Senthil-Nathan 2013, Poreddy et al. 2015). Of these, metabolic resistance is the most common mechanism that presents the greatest challenge (Després et al. 2007, Birnbaum et al. 2017). This resistance mechanism involves nonspecific enzymes that normally detoxify toxins, and include monooxygenases, oxidases, antioxidases, hydrolases, and transferases (Roy et al. 2016, Birnbaum et al. 2017). Insects exposed to toxins may possess higher levels or more efficient forms of these enzymes to induce tolerances to these toxins (Després et al. 2007, Tangtrakulwanich and Reddy 2014). For example, the detoxifying enzymes, including cytochrome P450s, UDP-glucuronosyltransferase, carboxylesterase, and glutathione-*S*-transferase, and the antioxidant enzymes, including, superoxide dismutase, catalase, and peroxidase, are activated after exposure to toxins (Mittapalli et al. 2007, Birnbaum et al. 2017, Wang et al. 2018). These rapid detoxification responses to toxins are vitally important for insect survival and reproduction.

*Calliptamus abbreviatus* Ikonn is an important grasshopper pest occurring in north Asia, with population outbreaks reducing alfalfa production in man-modified and natural grasslands (Tian 2003). Traditional control of *C. abbreviatus* relies mainly on the application of chemical insecticides, which might result in nontarget impacts, pesticide resistance, and environmental pollution (Wei et al. 2015). The effects of most botanical compounds on *C. abbreviatus* have not been documented. Because of the significant economic impact of *C. abbreviatus*, testing its response to a variety of botanical pesticides offers potential new ways to manage the pest populations.

We studied the survival, growth, and enzymatic activities of *C. abbreviatus* when exposed to six botanical toxins using a feeding trial and a field cage experiment. This research aimed to elucidate the response of *C. abbreviatus* to different botanical compounds to show their potential as biopesticides, which can be developed for the biological control of the grasshopper.

## Materials and Methods

### Ethics Statement

*Calliptamus abbreviatus* is a common insect pest in northern China and is not included in the ‘List of Protected Animals in China’. No specific permits were required for this study.

### *Calliptamus abbreviatus* Collection

We collected third-instar nymphs of *C. abbreviatus* using sweep nets from an alfalfa field (35.621°N, 118.369°E) in Linyi, Shandong Province, northeastern China. Vegetation in this area was dominated by alfalfa (*Medicago sativa* L) and is the preferred host for *C. abbreviatus*. The nymphs were temporarily maintained in metal frame cages and placed in an illuminated incubator for 1 d under an artificial light regime (13:11 [L:D] h) at 30 ± 1°C and a relative humidity of 75 ± 1%. Then, they were transferred to the artificial feeding trial as described below.

### Artificial Feeding Test of Six Plant-Derived Compounds

We studied the growth of *C. abbreviatus* when reared on food treated with the botanical compounds, rutin, quercetin, nicotine, matrine, azadirachtin, and rotenone. The compounds (>98% purity) were purchased from Sigma–Aldrich (St Louis, MO). A total of 700 third-instar females (starved for 24 h prior) were selected and 20 were randomly assigned to 35 plastic cages (30 × 20 × 10 cm^3^). Because the gender of the early instars is difficult to identify, third-instar female individuals were selected based on external morphology of the reproductive system. Alfalfa was used to feed the grasshopper nymphs. The plant was freshly collected from an alfalfa field and then treated with the six botanical compounds. We prepared a 0.01% solution (100 mg/l) of each compound using dimethyl sulfoxide (DMSO). A pure solution of DMSO was used as the control. For each cage, 100 ml of the prepared solution was evenly applied to 100 g of fresh *M. sativa* by a 200-ml hand sprayer, allowed to dry, and then provided to *C. abbreviatus*. The artificial feeding experiment included seven treatments, and each treatment was replicated five times. Treated fresh *M. sativa* in each cage was replaced every 24 h. Nymphs in the different treatments were maintained under an artificial light regime (13:11 [L:D] h) at a temperature of 30°C and relative humidity of 75%, with the bioassay lasting 7 d. We recorded the number of surviving insects in each cage and removed all dead individuals daily. Survival rate (%) was calculated as the number of individuals alive on the last day/number of initial individuals (20). At the end of the seventh day, a total of 15 female nymphs were collected (three females from each of five cages) from each treatment and used for enzyme activity analysis (see below). Other surviving individuals in each cage were euthanized by diethyl ether and dried at 90°C for 38 h. They were then individually weighed to determine the body dry mass (mg). Total 260 grasshopper individuals of all seven treatments were measured. Before the indoor feeding trial commenced, a cohort of 50 *C. abbreviatus* third-instar females were euthanized and dried by the same method, and a mean body mass (mg) of initial third-instar nymphs was determined. This served as baseline data for calculating the body mass increase. Increased body mass (mg) of each treatment was calculated by subtracting the basic third-instar body mass from the body mass on the final day of the feeding trials. Growth rate (mg/d) was calculated by body mass increase (mg)/developmental time (7 d).

### ELISA Analysis of *Calliptamus abbreviatus* Enzyme Activity

The rapid ELISA-based measurement described by Li et al. (2019) was used to detect the ROS level, and enzyme activities of CYP450s (cytochrome P450s), GSTs (glutathione-*S*-transferase), CarEs (carboxylesterase), UGTs (UDP-glucuronosyltransferase), SOD (superoxide dismutase), CAT (catalase), and POD (peroxidase) in grasshopper samples. The whole body of each female nymph collected from artificial feeding trial (see above) was used for rapid ELISA-based measurement. Briefly, each grasshopper individual was homogenized in 1-ml phosphate-buffered saline and disrupted by ultrasonication. Homogenates were centrifuged, and the supernatants were separated and used for analysis by ELISA kit (GTX, USA), according to the manufacturer instructions. All the ELISA reagents and samples were brought to room temperature for 30 min. We added 50 μl standard to each standard well, 50-μl sample to each sample well, and 50-μl sample diluent to each blank/control well. Then, 100 μl of horseradish peroxidase-conjugate reagent was added to each well and incubated for 60 min at 37°C. We used the undiluted Wash Buffer to wash the Microtiter Plate, and sequentially added 50-μl Chromogen Solution A and B to each well. The mixture was incubated at 37°C for 15 min in the dark, after which 50-μl Stop Solution was added. The optical density at 450 nm was read using a Microelisa Stripplate reader and used to calculate the ROS level or enzyme activities according to the constructed standard curve.

### Field Cage Study of Six Plant-Derived Compounds

We studied the survival rate of *C. abbreviatus* nymphs when reared on plants to which the six botanical compounds had been applied separately, in a field cage study. The cage trial was conducted on an alfalfa field in July 2018. We removed all other plant species in the field to ensure that only *M. sativa* remained. Then, the plant height and biomass were evaluated in five randomly selected quadrats (1 m × 1 m) using the same methods by Zhu et al. (2012). The *M. sativa* plants were mainly at the vegetative stage, with height ranging from ~47.6 to ~53.9 cm and dry biomass ranging from 95.6 to 103.3 g/m^2^.

A total of 35 screen cages (1 m × 1 m × 1 m) were installed in the *M. sativa* field, using iron rod frames covered with 1 mm^2^ cloth mesh. Each cage included ten *M. sativa*. Before adding *C. abbreviatus*, all potential natural enemies in these field cages were removed. A total of 700 third-instar *C. abbreviatus* female nymphs were assigned randomly to the 35 cages (20 individuals per cage). Preparations of 100 ml solutions of each botanical compound were applied evenly to *M. sativa* in cages using a 200-ml hand sprayer. A DMSO treatment was used as the control. This field cage experiment included seven treatments and five replications per treatment. We inspected the field cages daily to record survival and remove the dead individuals in each cage. The field cage study was conducted for 14 d. Survival rate (%) on days 7 and 14 was calculated as the number of surviving individuals/number of initial third-instar individuals.

### Data Analyses

Normality, homoscedasticity, and independence of errors of grasshopper variables were assessed, and all fit the ANOVA’s assumptions. One-way analysis of variance (ANOVA) and Turkey’s HSD were used to compare the survival rate, body mass, growth rate, and enzyme activity of grasshoppers among the treatments. We used SAS version 8.0 for all analyses at *P* < 0.05 significant level.

## Results

### *Calliptamus abbreviatus* Growth When Exposed to Six Botanical Compounds

**Fig. 1.**
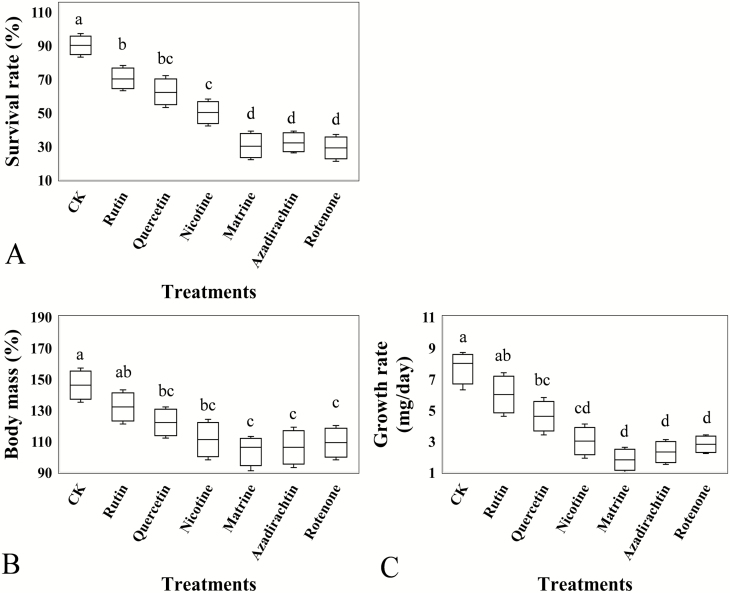
*Calliptamus abbreviates* survival rate (A, %), body mass (B, mg), and growth rate (C, mg/day) when exposed to six botanical compounds. CK represents the grasshoppers treated by pure solution of dimethyl sulfoxide. Boxes with different lowercase letters are significantly different based on Turkey’s HSD analysis at *P* < 0.05.

*Calliptamus abbreviatus* had significantly reduced survival (*F* = 16.92, df = 6, 28, *P* = 0.036, Fig. 1A) when exposed to the six botanical compounds in the feeding experiment. The body mass (*F* = 12.63, df = 6, 28, *P* = 0.043, Fig. 1B) and growth rate (*F* = 14.51, df = 6, 28, *P* = 0.041, Fig. 1C) were also significantly decreased except in the rutin treatment. Of the six compounds, matrine, azadirachtin, and rotenone had higher adverse effects on *C. abbreviatus* growth.

### ROS Level

**Fig. 2.**
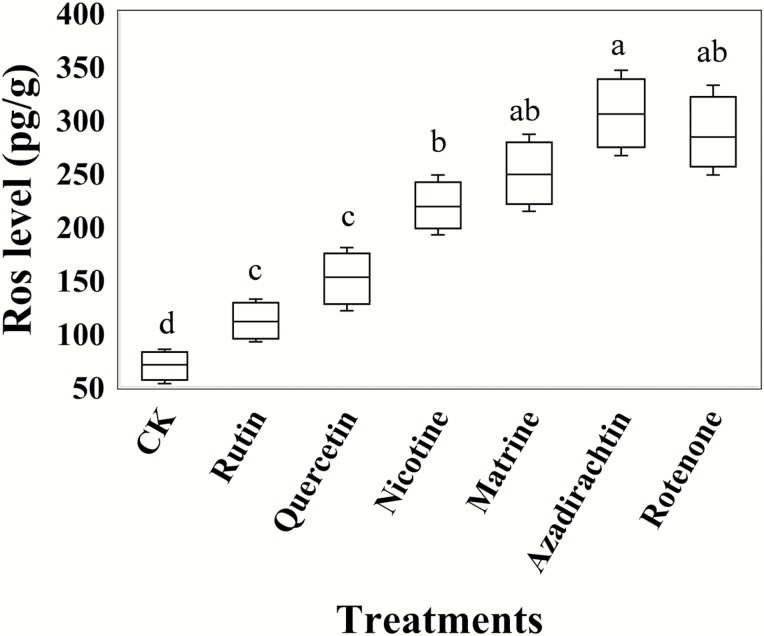
Reactive oxygen species (ROS) levels (pg/g) of *Calliptamus abbreviates* exposed to food treated with six botanical compounds. Boxes with different lowercase letters are significantly different based on Turkey’s HSD analysis at *P* < 0.05.

ROS levels in *C. abbreviatus* were detected by ELISA (Fig. 2). *Calliptamus abbreviatus* exhibited significantly (*F* = 21.27, df = 6, 28, *P* = 0.008) increased ROS levels when exposed to the six plant compounds. Exposure to nicotine, matrine, azadirachtin, and rotenone induced the highest ROS levels in *C. abbreviatus*.

### Detoxifying Enzymes Activity

**Fig. 3.**
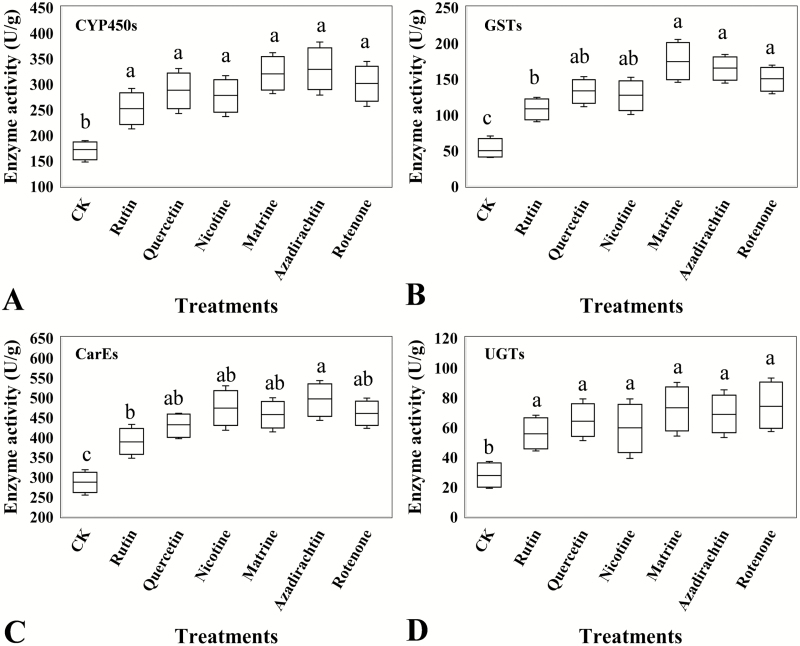
Detoxification enzyme activity of *Calliptamus abbreviatus* exposed to food treated with different xenobiotic compounds. (A) CYP450s, cytochrome P450s (U/g); (B) GSTs, glutathione-*S*-transferase (U/g); (C) CarEs, carboxylesterase (U/g); and (D) UGTs, UDP-glucuronosyltransferases (U/g). CK represents the grasshoppers treated by pure solution of dimethyl sulfoxide. Boxes with different lowercase letters are significantly different (*P* < 0.05) based on Turkey’s HSD analysis.

The ELISA analysis demonstrated that the activities of the detoxifying enzyme, CYP450s (*F* = 16.19, df = 6, 28, *P* = 0.017, Fig. 3A), GSTs (*F* = 12.07, df = 6, 28, *P* = 0.041, Fig. 3B), CarEs (*F* = 12.81, df = 6, 28, *P* = 0.048, Fig. 3C), and UGTs (*F* = 14.94, df = 6, 28, *P*= 0.025, Fig. 3D), were significantly induced in *C. abbreviatus* when it was exposed to the six botanical compounds. Treatment with matrine, azadirachtin, and rotenone produced a higher GST activity than the rutin treatment.

### Antioxidant Enzymes Activity

**Fig. 4.**
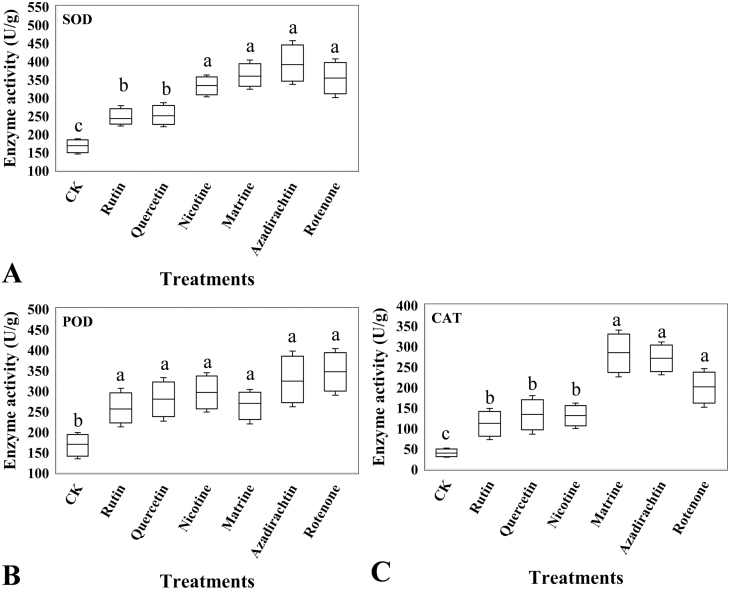
Antioxidant enzymes activity of *Calliptamus abbreviates* exposed to food treated with six different botanical compounds. (A) SOD, superoxide dismutase (U/g); (B) POD, peroxidase (U/g); and (C) CAT, catalase (U/g). CK represents the grasshoppers treated by pure solution of dimethyl sulfoxide. Boxes with different lowercase letters are significantly different (*P* < 0.05) based on Turkey’s HSD analysis.

The ELISA analysis showed that the activities of the antioxidant enzymes, SOD (*F* = 18.67, df = 6, 28, *P* = 0.009, Fig. 4A), CAT (*F* = 12.29, df = 6, 28, *P* = 0.040, Fig. 4B), and POD (*F* = 15.95, df = 6, 28, *P* =0.016, Fig. 4C), were significantly increased in *C. abbreviatus* when it was exposed to the six botanical compounds. Matrine, azadirachtin, and rotenone treatments had higher SOD, CAT, and POD activity compared with rutin and quercetin treatments.

### *Calliptamus abbreviates* Survival in Field Cages When Exposed to Botanical Compounds

**Fig. 5.**
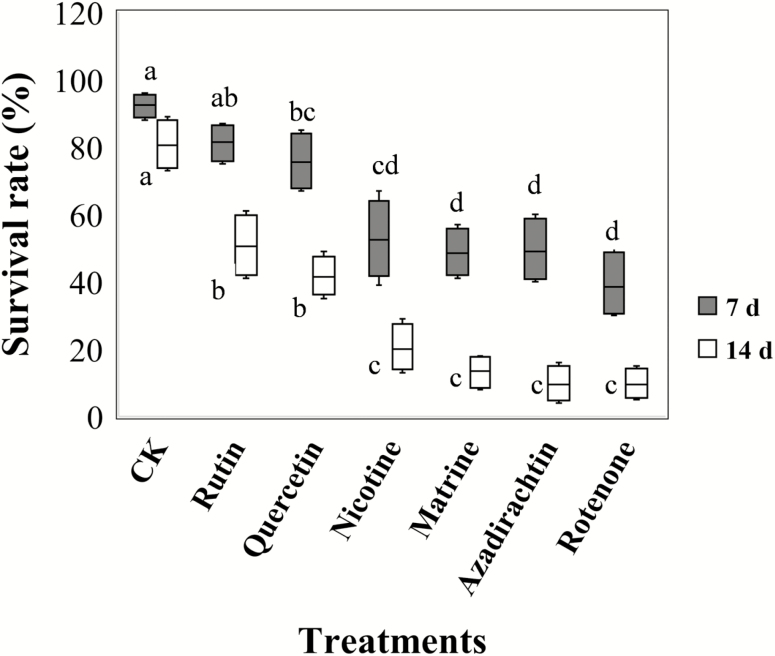
*Calliptamus abbreviatus* survival rate (%) at 7 and 14 d when exposed to six xenobiotic compounds within field cages. CK represents the grasshoppers treated by pure solution of dimethyl sulfoxide. Boxes with different lowercase letters within the same observation day are significantly different based on Turkey’s HSD analysis at *P* < 0.05.

A 14-d field cage study determined the effects of the six botanical compounds on *C. abbreviatus* survival (Fig. 5). The survival rate significantly decreased (*F* = 29.57, df = 6, 28, *P* < 0.001) at 7 d when *C. abbreviatus* fed on plants to which the botanical toxins were applied, except the rutin treatment. At 14 d, all compounds had produced significant (*F* = 35.61, df = 6, 28, *P* < 0.001) adverse effects on *C. abbreviatus*, and the survival rates were significantly (*F* = 39.86, df = 6, 28, *P* < 0.001) lower than at 7 d. The nicotine, matrine, azadirachtin, and rotenone treatments induced greater mortality compared with the rutin or quercetin treatments.

## Discussion

Studies on botanical compounds could provide new options for biopesticide development, which can have potential applications in pest control (Wu and Baldwin 2010, Richards et al. 2016, Goldar et al. 2018). In the present study, rutin, quercetin, nicotine, matrine, azadirachtin, and rotenone, all exhibited suppressive effects on the growth and survival of third-instar female nymphs of *C. abbreviatus*. This indicated that all these compounds possessed toxic properties and could potentially be used to control *C. abbreviatus* nymphs. These results are consistent with many previous studies (Mesbah et al. 2007, Senthil-Nathan 2013, Taggar and Gill 2016, Khan et al. 2017), which have shown that insect growth is often negatively correlated with the levels of toxic plant compounds. However, the level of the adverse effects of the six compounds on *C. abbreviatus* varied. Matrine, azadirachtin, and rotenone exhibited greater toxicity on *C. abbreviatus* growth and survival, compared with the other compounds.

Herbivorous insects have evolved multiple mechanisms to cope with toxic compounds (Caballero et al. 2008). Studies on these mechanisms can provide insight into the success of applied new botanical pesticides (Celorio-Mancera et al. 2011, Herde and Howe 2014, Erb and Robert 2016). Insect resistance to toxins generally results from the overproduction of detoxification-related enzymes, such as CYP450s, GSTs, CarEs, and UGTs, that can metabolize toxins (Roy et al. 2016). Previous studies showed that the CYP450s could catalyze monooxygenase reactions, and this enzyme activity increased significantly in *Bombyx mori* and *Helicoverpa armigera* larvae after treatment with quercetin (Despres et al. 2007, Chen et al. 2017). The overproduction of GSTs could conjugate substrates with reduced glutathione, and these were induced significantly by toxic pyrethroids or glucosinolates in the aphid *Myzus persicae* (Francis et al. 2005). The UGTs can generate water-soluble products and were involved in the degradation of botanical toxins in *Bombyx mori* and *Manduca sexta* (Luque et al. 2002). Aphids and mosquitoes treated with toxins had higher productions of CarEs than untreated ones (Tangtrakulwanich and Reddy 2014). In the present study, we also found that *C. abbreviatus* third-instar nymphs had significantly increased enzyme activities of these four detoxification enzymes when exposed to the six botanical compounds.

Secondary plant compounds, as toxic stimuli, can also result in the production of ROS (Apel and Hirt 2004, Huang et al. 2013, Matsumura et al. 2017). The increased toxic species of oxygen in aerobic organisms generally cause oxidative damage leading to programed cell death or apoptosis (Brosché et al. 2009, Dubose et al. 2009, Huang et al. 2013, Klumpen et al. 2017). An insect defense mechanism to reduce oxidative damage is the upregulation of antioxidant enzymes including SOD, CAT, and POD (Aucoin et al. 1991, Mittapalli et al. 2007, Lee and Berenbaum 2010). For example, the activities of these antioxidant enzymes were significanly increased to resist ROS increase in lepidopteran larvae feeding on azadirachtin and hypericin (Lee and Berenbaum 2010, Huang et al. 2013). In the present study, ROS concentration increased significantly in *C. abbreviatus* third-instar nymphs when exposed to the six botanical compounds. The high ROS level resulted in increased level of oxidative damage in *C. abbreviatus*. This may explain why *C. abbreviatus* nymphs had significantly reduced growth and low survival when exposed to the six compounds. Of the six compounds, the ROS level in the matrine, azadirachtin, and rotenone treatments was higher than in the rutin and quercetin treatments. This indicated that the former compounds were more toxic to *C. abbreviatus* third-instar nymphs than the latter compounds. We found that the botanical compounds induction of an elevated ROS significantly increased the levels of the antioxidant enzymes, SOD, POD, and CAT. Induction of an array of robustly active antioxidant enzymes could allow *C. abbreviatus* nymphs to reduce oxidative damage.

Induced insect resistance to botanical toxins comes at the expense of increased energetic costs (Termonia et al. 2001, Despres et al. 2007, Birnbaum et al. 2017). The significantly increased activities of detoxifying and antioxidant enzymes in *C. abbreviatus* imply that survival, following exposure to the botanical compounds, required considerable energy consumption and resulted in reduced phenotypic parameters (Whitman and Ananthrakrishnan 2009, Castañeda et al. 2010), such as the decreased size and reduced growth rate. This may be another reason why *C. abbreviatus* nymphs had reduced growth when challenged with the botanical compounds. This response may at first appear to be detrimental to *C. abbreviatus* nymphs, but it may be beneficial if it permits the individual to survive poisoning stress. In addition, the enhancing activity of the detoxifying and antioxidant enzymes may also be the consequence of altered genes or pathways regulating these enzymes (Despres et al. 2007, Roy et al. 2016). How the six botanical compounds influence such genes or pathways (molecular mechanisms) is unclear and should be addressed in future studies.

We studied the effects of six botanical compounds on *C. abbreviatus* growth performance and enzyme activities. All six compounds produced toxic effects, especially matrine, azadirachtin, and rotenone. These compounds inhibited the growth of *C. abbreviatus* and significantly reduced the survival rate. The detrimental effects on *C. abbreviatus* suggest that the compounds could be developed into bioinsecticides for grasshopper control. However, we only compared the response of the grasshopper to six botanical compounds at 0.01% dose. The lethal dose of 50% (LD_50_), lethal concentration of 50% (LC_50_), and lethal time of 50% (LT_50_) of the six compounds to *C. abbreviatus* should be studied in the future. In addition, these compounds may also have toxic activity to nontarget invertebrates. Considerable formulation and regulatory work would also be necessary. For example, many studies have demonstrated that the toxicity of botanical compounds can be enhanced in mixtures (Koul et al. 2004, Singh et al. 2009, Tangtrakulwanich and Reddy 2014). Introducing new control technologies of insect pest does not require abandoning traditional methods. Naturally derived compounds are unlikely to solve all pest control problems and therefore could be integrated with traditional ones. One example would be the use of the botanical compounds in this study as adjuvants in mixtures with traditional insecticides.
